# *PIK3CA* mutations are frequent in esophageal squamous cell carcinoma associated with chagasic megaesophagus and are associated with a worse patient outcome

**DOI:** 10.1186/s13027-018-0216-3

**Published:** 2018-12-29

**Authors:** Fernanda Franco Munari, Adriana Cruvinel-Carloni, Croider Franco Lacerda, Antônio Talvane Torres de Oliveira, Cristovam Scapulatempo-Neto, Sandra Regina Morini da Silva, Eduardo Crema, Sheila Jorge Adad, Maria Aparecida Marchesan Rodrigues, Maria Aparecida Coelho Arruda Henry, Denise Peixoto Guimarães, Adhemar Longatto-Filho, Rui Manuel Reis

**Affiliations:** 1 Molecular Oncology Research Center, Barretos Cancer Hospital, Rua Antenor Duarte Villela, 1331, Barretos, SP CEP 14784 400 Brazil; 2 Department of Digestive Surgery, Barretos Cancer Hospital, Barretos, SP Brazil; 3 Department of Pathology, Diagnosis of Biopsies and Surgical Specimens, Barretos Cancer Hospital, Barretos, SP Brazil; 4 Department of Digestive Surgery and Pathology, Medical School, UFTM – Federal University of Triangulo Mineiro, Uberaba, Minas Gerais Brazil; 5 Departament of Gastroenterology Surgery and Pathology, Medical School, UNESP, São Paulo State University, Botucatu, SP Brazil; 6 Department of Endoscopy, Barretos Cancer Hospital, Barretos, SP Brazil; 7 Department of Radiology and Oncology, Medical School, USP - University of São Paulo, São Paulo, Brazil; 8 Medical Laboratory of Medical Investigation (LIM) 14, Department of Pathology, Medical School, USP - University of São Paulo, São Paulo, Brazil; 9 Life and Health Sciences Research Institute (ICVS), School of Health Sciences, University of Minho, Braga, Portugal; 10 ICVS/3B’s - PT Government Associate Laboratory, Braga/Guimarães, Portugal

**Keywords:** Esophageal cancer, *Trypanosoma cruzi*, Achalasia, Esophageal squamous cell carcinoma, Chagasic megaesophagus, *PIK3CA*, Mutation

## Abstract

**Background:**

Chronic diseases such as chagasic megaesophagus (secondary to Chagas’ disease) have been suggested as etiological factors for esophageal squamous cell carcinoma; however, the molecular mechanisms involved are poorly understood.

**Objective:**

We analyzed hotspot *PIK3CA* gene mutations in a series of esophageal squamous cell carcinomas associated or not with chagasic megaesophagus, as well as, in chagasic megaesophagus biopsies. We also checked for correlations between the presence of *PIK3CA* mutations with patients’ clinical and pathological features.

**Methods:**

The study included three different groups of patients: i) 23 patients with chagasic megaesophagus associated with esophageal squamous cell carcinoma (CM/ESCC); ii) 38 patients with esophageal squamous cell carcinoma not associated with chagasic megaesophagus (ESCC); and iii) 28 patients with chagasic megaesophagus without esophageal squamous cell carcinoma (CM). *PIK3CA* hotspot mutations in exons 9 and 20 were evaluated by PCR followed by direct sequencing technique.

**Results:**

*PIK3CA* mutations were identified in 21.7% (5 out of 23) of CM/ESCC cases, in 10.5% (4 out of 38) of ESCC and in only 3.6% (1 case out of 28) of CM cases. In the CM/ESCC group, *PIK3CA* mutations were significantly associated with lower survival (mean 5 months), when compared to wild-type patients (mean 2.0 years). No other significant associations were observed between *PIK3CA* mutations and patients’ clinical features or *TP53* mutation profile.

**Conclusion:**

This is the first report on the presence of *PIK3CA* mutations in esophageal cancer associated with chagasic megaesophagus. The detection of *PIK3CA* mutations in benign chagasic megaesophagus lesions suggests their putative role in esophageal squamous cell carcinoma development and opens new opportunities for targeted-therapies for these diseases.

## Introduction

Esophageal cancer is the eighth most frequent type of cancer in the world and the sixth most lethal, occurring mainly in developing countries such as Brazil [1]. The most frequent histological subtype is esophageal squamous cell carcinoma (ESCC), accounting for 90% of cases, especially in high-risk areas [2]. The main risk factors for ESCC are alcohol consumption, tobacco (mainly in association) and hot-beverage consumption [3]. It is also reported that chronic diseases, such as the chagasic megaesophagus, can be associated with ESCC development [4].

Chagasic megaesophagus is the late manifestation of Chagas’ disease (caused by the protozoan *Trypanosoma cruzi*) [5]. Direct infection of *Trypanosoma cruzi* will lead to destruction of the intramural myenteric neurons in the esophagus, causing inflammation and production of neurotoxins. This will result in uncoordinated contractions and reduction of peristalsis of the organ, altering the functioning of the lower esophageal sphincter and progressive dilation of the esophagus (megaesophagus) [6]. In Brazil, one of the endemic regions of Chagas’ disease, approximately 4 million people are infected with the parasite and about 6–7% of these patients will develop chagasic megaesophagus [5]. Patients affected with this lesion are more likely to develop ESCC (3–10%) when compared to the general population [4].

The carcinogenic mechanisms of ESCC development in the context of chagasic megaesophagus have been little explored. Recently, our group showed the high frequency (13/32, 40.6%) of *TP53* mutations in ESCC associated with chagasic megaesophagus [7]. Moreover, we also reported the presence of microsatellite instability (MSI) in a small fraction (1/19, 5.3%) of cases [8]. However, many other genes are known to be involved in ESCC carcinogenesis as demonstrated by the TCGA consortium [9].

One of these genes is the *PI3KCA*, which encodes the protein phosphatidylinositol 3-kinase (PI3K), that belongs to a family of lipid kinases that encodes the p110α catalytic subunit [10]. PI3K is a quite complex signaling pathway since it regulates cell growth, proliferation, cell motility, the production of new proteins, apoptosis and cell survival [10]. Therefore, its activation will lead to many downstream pathways that regulate several cellular functions, including those involved in the development of cancer [10, 11]. Recurrent *PI3KCA* oncogenic mutations were identified in several types of tumors, including colorectal, breast, ovary, gastric, and recently in ESSC [12]. The mutations occur mainly in exons 9 (E542K and E545K) and 20 (H1047R) [12]. Recently, it was shown that *PIK3CA* mutations, namely H1047R, also disrupt cellular genetic stability, increasing the frequency of chromosomal errors and leading to tetraploidy [13]. Importantly, therapeutic strategies targeting the PIK3/Akt signaling pathway have been developed and could constitute effective treatment options for patients harboring *PI3KCA* mutations [14].

Therefore, in the current study we performed the mutation analysis of *PIK3CA* gene in patients with ESCC and chagasic megaesophagus associated or not with ESCC, and searched for associations between the mutation status and patients’ clinical and pathological features.

## Materials and methods

### Study population

In this retrospective study, we analyzed 89 formalin-fixed paraffin-embedded (FFPE) tissues of three groups of patients: i) 23 patients with chagasic megaesophagus associated with esophageal squamous cell carcinoma (CM/ESCC); ii) 38 patients with esophageal squamous cell carcinoma without chagasic megaesophagus (ESCC); and iii) 28 patients with chagasic megaesophagus without esophageal squamous cell carcinoma (CM). All chagasic megaesophagus patients were serologic positive for Chagas’ disease and/or had exams (imaging and histopathology) that confirmed the presence of megaesophagus. The patients with esophageal squamous cell carcinoma without chagasic megaesophagus were all serologic negative for Chagas’ disease and had exams (imaging and histopathology) that confirmed malignant disease. These patients were previously described for their clinical-pathological and molecular *TP53* and MSI features [7, 8].

The samples were obtained from patients treated between 1990 and 2016 in three different institutions from the Southeast of Brazil, namely: Barretos Cancer Hospital, Barretos, São Paulo State; Federal University of Triângulo Mineiro (UFTM), Uberaba, Minas Gerais State and São Paulo State University (UNESP), Botucatu, São Paulo State. All clinical and pathological information was obtained through medical record review.

### DNA isolation

Following tissue macro-dissection, DNA was isolated from FFPE tissue representative of the tumor lesions in ESCC and CM/ESCC groups and esophageal tissues in CM group, as previously described [7]. Briefly, the tumor area was delineated in a hematoxylin-eosin stained (HE, Merck KGaA, GE) section by a pathologist, and the marked area was scraped by scalp from 3 to 5 10 μm unstained slides into a 1,5 ml tube. Afterwards, the tissue was subjected to the dewaxing step by heating (80 °C – 20 min), followed by sequential washing in xylol (5 min) and decreasing concentrations of ethanol (1 min - 100, 70 and 50%) and nuclease-free water9 (1 min). DNA isolation was performed using the QIAamp DNA Micro Kit (Qiagen) following the manufacture’s protocol.

### *PIK3CA* mutation analysis

Polymerase chain reaction (PCR) followed by direct sequencing (Sanger method) was performed for the analysis of hotspot mutations (exons 9 and 20) of the *PIK3CA* gene as previously described [15]. The PCR was performed on the 89 samples under the following conditions: 5X Flexi Buffer (pH 8.5) and 25 mM MgCl2 (Promega, USA), 200 μM dNTPmix (Invitrogen, USA), 200 nM primers exon 9 (forward 5’-CTGTGAATCCAGAGGGGAAA-3′ and reverse 5’-ACATGCTGAGATCAGCCAAAT-3′) and exon 20 (forward 5’-ATGATGCTTGGCTCTGGAAT-3′ and reverse 5’-GGTCTTTGCCTGCTGAGAGT-3′), 1.25 U GoTaq®Hot Start Polymerase (Promega, USA), and nuclease-free water (Gibco, BRL, Life Technologies, USA) in a final volume of 25 μl and 5 μl of DNA at 50 ng/μl from each patient were added [15]. Amplification was performed in a thermocycler according to the protocol: 96 °C for 15 min, followed by 40 cycles at 96 °C for 45 s, 55.5 °C for 45 s and 72 °C for 45 s and final extension of 72 °C for 10 min, followed by a hold at 4 °C. PCR products were subjected to 1.5% agarose gel electrophoresis with Gel Red (Biotium, Hayward, CA) to evaluate the amplification of the gene of interest.

After agarose gel validation, we purified the preparation using the enzyme ExoSap-IT (GE Technology, Cleveland, USA), followed by the sequencing reaction using BigDye Terminator v3.1 (Applied Biosystems, USA) and 3.2 μM of specific primers and re-purified with xTerminator (Life Technology). The products were sequenced using the 3500 series Genetic Analyzer Capillary Sequencer (Applied Biosystems, USA). All the cases that showed mutations were confirmed with a new PCR reaction and direct sequencing.

### Statistical analysis

Characterization of the study population was performed through frequency tables for qualitative variables, and measures of central tendency and dispersion (mean, standard deviation, minimum and maximum) for the quantitative variables, comparing the different groups. To verify the association between *PIK3CA* mutation status and clinical groups, pathological and molecular features, Chi-square or Fisher’s exact tests were applied. We performed an overallsurvival analysis using the Kaplan-Meier limit estimator and the Log-rank test to compare the survival curves between the groups.

The level of significance adopted was 5% (*p* ≤ 0.05). Statistical analyzes were in SPSS software v.21.0.

## Results

### Characterization of the population

The clinical-pathological characteristics of the patients in the three groups are described in Table 1. The mean age of the patients was higher in the chagasic groups (Table 1). As already reported in our previous studies [7], concerning risk factors for esophageal cancer, the ESCC and CM/ESCC groups were statistically associated with higher tobacco and alcohol consumption (Table 1).

**Table 1:** The clinical-pathological features of the three groups of patients

Variable		Groups (*n* = 89)			
	Category	CM/ESCC (*n* = 23)	ESCC (*n* = 38)	CM (*n* = 28)	*p*-value
Gender	Female	4 (17.4%)	7 (18.4%)	4 (14.3%)	0.937^**^
	Male	19 (82.6%)	31 (81.6%)	24 (85.7%)	
Age (years)	Mean (SD)	59 (11)	57 (9)	59 (11)	**0.039** ^***^
	Min - Max	37–76	36–75	37–76	
Alcohol consumption	No	7 (31.8%)	8 (21.1%)	20 (71.4%)	**< 0.001** ^*^
	Yes	15 (68.2%)	30 (78.9%)	8 (28.6%)	
	Missing	11	0	0	
Tobacco consumption	No	4 (18.2%)	6 (15.8%)	16 (57.1%)	**< 0.001** ^*^
	Yes	18 (81.8%)	32 (84.2%)	12 (42.9%)	
	Missing	1	0	0	
Tumor differentiation	Well differentiated	5 (22.7%)	7 (19.4%)	NA	0.639^**^
	Moderate differentiated	16 (72.7%)	24 (66.7%)	NA	
	Poorly differentiated	1 (4.5%)	5 (13.9%)	NA	
	Missing	1	2	NA	
TNM Staging	I/II	4 (23.5%)	16 (42.1%)	NA	0.235^*^
	III/IV	13 (76.5%)	22 (57.9%)	NA	
	Missing	6	0	NA	
Megaesophagus grades	GI/GII	10 (45.5%)	NA	4 (14.3%)	**0.025** ^*^
	GIII/GIV	12 (54.5%)	NA	24 (85.7%)	
	Missing	1	NA	0	

^*^Chi-square association test; ^**^Fisher’s exact test; ^***^Analysis of variance. CM/ESCC – chagasic megaesophagus associated with esophageal squamous cell carcinoma; ESCC – esophageal squamous cell carcinoma without chagasic megaesophagus; CM – chagasic megaesophagus without esophageal squamous cell carcinoma; SD - standard deviation; NA – not applicable; Bold numbers - statistical significance

### Mutation analysis of *PIK3CA* gene

**Fig. 1:**
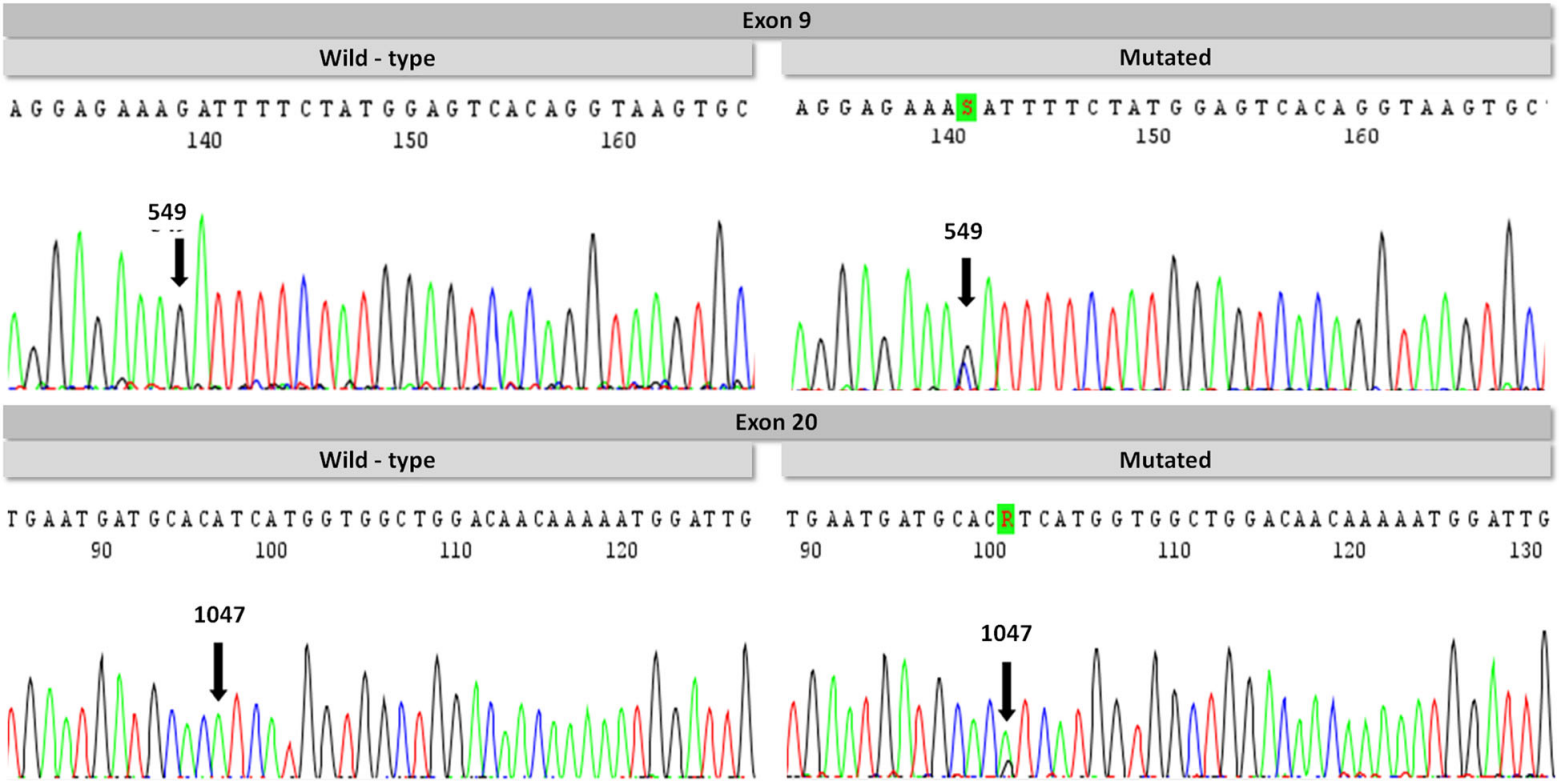
Electropherogram of *PIK3CA* gene. Exon 9 – wild-type sequence and mutated sequence (D549H). Exon 20 – wild-type sequence and mutated sequence (H1047R)

**Fig. 2:**
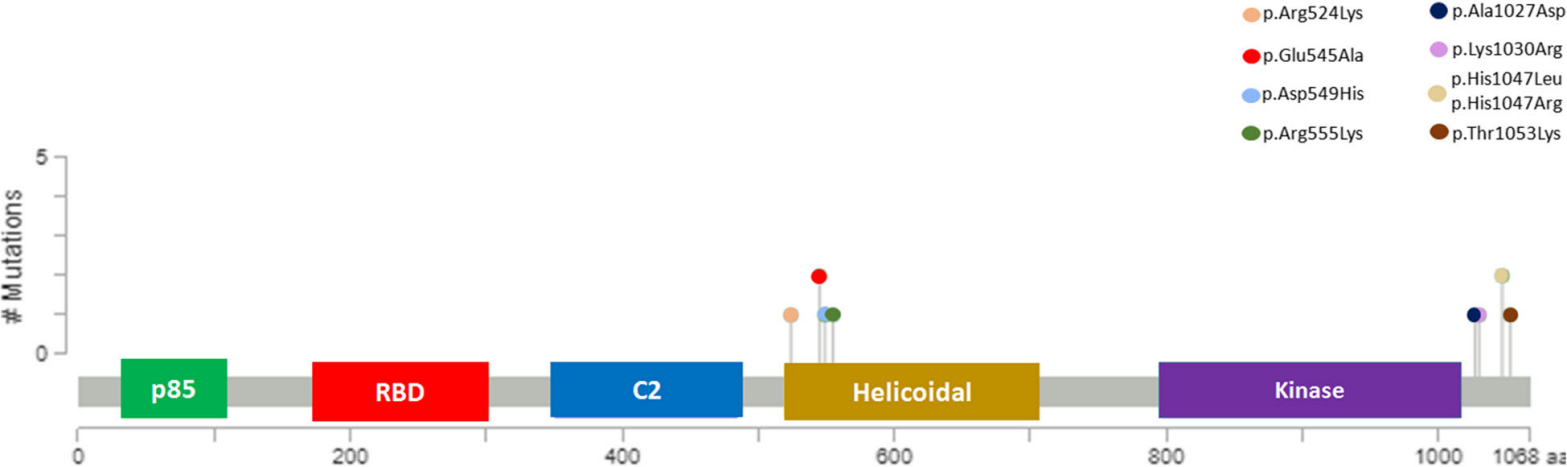
*PIK3CA* protein and missense mutation overview

The *PIK3CA* mutation analysis showed the presence of mutations in 21.7% of patients in CM/ESCC group, followed by 10.5% in ESCC group and 3.6% in CM group (Fig. 1 and Table 2). The frequency of mutations was similar in exons 9 and 20 (Table 3). With the exception of three variants (A1027D, K1030R and T1053K), all the other mutations have already been reported in the Catalogue of Somatic Mutations in Cancer database – COSMIC (http://cancer.sanger.ac.uk/cosmic) (Fig. 2 and Table 3).

**Table 2:** Frequency of *PIK3CA* mutations in the three study groups

Variable		Groups (*n* = 89)			
	Category	CM/ESCC (*n* = 23)	ESCC (*n* = 38)	CM (*n* = 28)	*p*-value
*PIK3CA* gene	WT	18 (78.3%)	34 (89.5%)	27 (96.4%)	0.132^**^
	MUT	5 (21.7%)	4 (10.5%)	1 (3.6%)	

^**^Fisher’s exact test. CM/ESCC – chagasic megaesophagus associated with esophageal squamous cell carcinoma; ESCC – esophageal squamous cell carcinoma without chagasic megaesophagus; CM – chagasic megaesophagus without esophageal squamous cell carcinoma. WT – wild-type; MUT – mutant; N – number of cases

**Table 3:** Profile of oncogenic *PIK3CA* mutations in the three study groups

Group	Sample ID	Exon	Codon	Codon (WT – MUT)	Type of mutation	Amino acids change	Nature of mutation	COSMIC ID
CM/ESCC	111 T	9	545	GAG – GCA	A → C	E545A	Missense	COSM12458
	122 T	9	549	GAT – CAT	G → C	D549H	Missense	COSM219119
	114 T	20	1027	GCC – GAC	C → A	A1027D	Missense	Not reported
	119 T	20	1047	CAT – CTT	A → T	H1047L	Missense	COSM776
	124 T	20	1047	CAT–CGT	A → G	H1047R	Missense	COSM775
ESCC	26 T	9	545	GAG – GCG	A → C	E545A	Missense	COSM12458
	120 T	9	555	AGG – AAG	G → A	R555K	Missense	COSM1716158
	36 T	9	524	AGG – AAG	G → A	R524K	Missense	COSM53245
	4 T	20	1030	AAA – AGA	A → G	K1030R	Missense	Not reported
CME	101 M	20	1053	ACA – AAA	C → A	T1053K	Missense	Not reported

CME/ESCC – chagasic megaesophagus associated with esophageal squamous cell carcinoma; ESCC – esophageal squamous cell carcinoma without chagasic megaesophagus; CME – chagasic megaesophagus without esophageal squamous cell carcinoma. A – adenine; C – cytosine; G – guanine; T – thymine. WT – wild-type; MUT – mutant

**Fig. 3:**
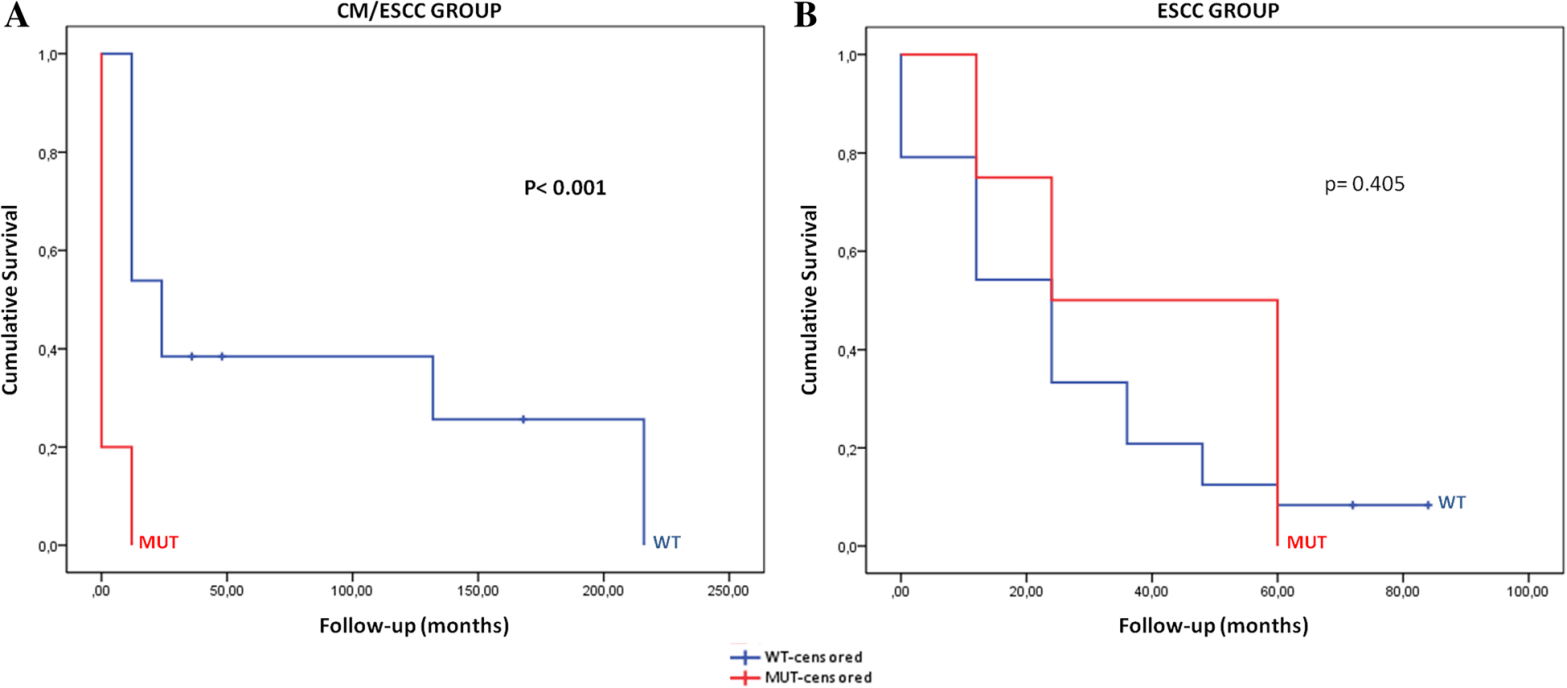
Cumulative survival of patients associated with the *PIK3CA* gene status. The red curves represent patients with mutation and the blue curves represent wild-type patients. **a** CM/ESCC – chagasic megaesophagus associated with squamous cell carcinoma of the esophagus; **b** ESCC – squamous cell carcinoma of the esophagus; MUT – mutant; WT – wild-type

No significant associations were observed between the *PIK3CA* mutation status and patients pathological features (Table 4). Furthermore, we assessed the role of *PIK3CA* mutations on patients’ overall survival in both groups affected by cancer (CM/ESCC and ESCC) (Fig. 3). In CM/ESCC group, we observed that the presence of *PIK3CA* mutations was significantly associated with a lower survival rate from the diagnosis of cancer compared to wild-type patients (Fig. 3a). The mean patients’ overall survival in cases from the CM/ESCC group mutated for the *PIK3CA* was 5 months, in comparison with 2.0 years for wild-type *PIK3CA* patients (Log-rank, *p* < 0.001) (Table 5).

**Table 4:** Association between *PIK3CA* mutation status with main clinical-pathological features in the three groups

Variable		*PIK3CA* gene								
		CM/ESCC group			ESCC group			CM group		
	Category	WT	MUT	p-value	WT	MUT	p-value	WT	MUT	*p*-value
Alcohol consumption	No	6 (35.3%)	1 (20%)	1.000^**^	7 (21.2%)	1 (20%)	1.000^**^	19 (70.4%)	1 (100%)	1.000^**^
	Yes	11 (64.7%)	4 (80%)		26 (78.8%)	4 (80%)		8 (29.6%)	0 (0%)	
Tobacco consumption	No	4 (23.5%)	0 (0%)	0.535^**^	5 (15.2%)	1 (20%)	1.000^**^	15 (100%)	1 (100%)	1.000^**^
	Yes	13 (76.5%)	5 (100%)		28 (84.8%)	4 (80%)		12 (44.4%)	0 (0%)	
Tumor differentiation	Well	4 (22.2%)	1 (25%)	1.000^**^	7 (22.6%)	0 (0%)	0.171^**^	NA	NA	NA
	Moderate	13 (72.2%)	3 (75%)		21 (67.7%)	3 (60%)		NA	NA	
	Poorly	1 (5.6%)	0 (0%)		3 (9.7%)	2 (40%)		NA	NA	
TNM Staging	I e II	4 (28.6%)	0 (0%)	0.541^**^	15 (46.9%)	1 (20%)	0.421^**^	NA	NA	NA
	III e IV	10 (71.4%)	3 (100%)		17 (53.1%)	4 (80%)		NA	NA	
Megaesophagus degree	GI/GII	7 (41.2%)	3 (60%)	0.406^**^	NA	NA	NA	3 (11.1%)	1 (100%)	0.143^**^
	GIII/GIV	10 (58.8%)	2 (40%)		NA	NA		24 (88.9%)	0 (0%)	
*TP53* gene [7]	WT	9 (50%)	2 (40%)	1.000^**^	20 (60.6%)	2 (40%)	0.632^**^	26 (96.3%)	1 (100%)	1.000^**^
	MUT	9 (50%)	3 (60%)		13 (39.4%)	3 (60%)		1 (3.7%)	0 (0%)	

^**^Fisher’s exact test; ^***^Analysis of variance. CME/ESCC – chagasic megaesophagus associated with esophageal squamous cell carcinoma; ESCC – esophageal squamous cell carcinoma without chagasic megaesophagus; CME – chagasic megaesophagus without esophageal squamous cell carcinoma. N – number of cases; SD – standard deviation; NA – not applicable; WT – wild-type; MUT – mutated

**Table 5:** The time and average of patients’ overall survival according to *PIK3CA* mutation status

Groups				Time					
	Variable	Total	N events	6 months	1 year	3 years	5 years	Median survival	*p*-value
CM/ESCC	WT	13	10	79.4%	72.2%	43.3%	28.9%	2 years	**< 0.001** ^*^
	MUT	5	5	20.0%	0.0%	0.0%	0.0%	5 months	
ESCC	WT	24	22	82.6%	64.7%	32.6%	14.5%	2 years	0.405
	MUT	4	4	80.0%	80.0%	60.0%	0.0%	2.5 years	

^*^Log-rank test. CM/ESCC – chagasic megaesophagus associated with esophageal squamous cell carcinoma; ESCC – esophageal squamous cell carcinoma without chagasic megaesophagus; WT – wild-type; MUT – mutated; Bold numbers - statistical significance

Additionally, we evaluated the association of *PIK3CA* and *TP53* mutation status, and no association was found (Table 4).

## Discussion

Among the several risk factors for the development of ESCC, the chagasic megaesophagus (late complication of Chagas’ disease) has been a minor etiological factor and little explored [4]. Nevertheless, Chagas’ disease is still an important public health problem, particularly in Latin-America, where approximately 20 million people are infected with Chagas’ disease and approximately 6–7% of these people will develop chagasic megaesophagus [5, 16].

In the present study, we investigated the frequency of *PIK3CA* mutations in regions of hotspot (exons 9 and 20) in patients with chagasic megaesophagus associated with esophageal squamous cell carcinoma (CM/ESCC) and compared with patients with esophageal squamous cell carcinoma without chagasic megaesophagus (ESCC) and patients with chagasic megaesophagus without esophageal squamous cell carcinoma (CM). We observed that patients in the CM/ESCC group had a higher frequency of mutations (5/23, 21.7%) followed by patients in the ESCC group (4/38, 10.5%), and in the CM group (1/28, 3.6%). This is the first report of *PIK3CA* mutation in ESCC that developed in the context of chagasic megaesophagus and the significant frequency of mutations (~ 22%) suggest that *PIK3CA* plays an important role in the carcinogenesis of CM/ESCC patients. Moreover, the presence of *PIK3CA* mutation in a benign lesion further supports the putative role of chagasic megaesophagus as an ESCC-related condition. The frequency of mutations identified in our study is in line with that reported in the literature for ESCC patients, with frequencies varying from 2.2 to 32.8% (Table 6) [9, 17–33]. This variation can be due to several factors, such as type of tissue (frozen vs FFPE), distinct methodologies for mutation screening and distinct ethnic groups of patients. (Table 6).

**Table 6:** Frequency of *PIK3CA* mutations identified in patients with esophageal squamous cell carcinoma worldwide

References	Year	Country (Region)	Patients of study	*PIK3CA* mutated (%)	Type of sample	Techniques
Mori et al. [24]	2008	Japan	88	2.2%	FT	Direct Sequencing
Wang et al. [30]	2013	China	76	3.9%	FT	Direct Sequencing
Akagi et al. [17]	2009	Japan	52	7.7%	FFPE	Direct Sequencing
Kim et al. [21]	2016	Korea	534	10.5%	FFPE	Direct Sequencing
Phillips et al. [25]	2006	Australia	35	11.8%	FFPE	Direct Sequencing
Zheng et al. [33]	2016	China	79	19.7%	FT	Pyrosequencing
Shigaki et al. [27]	2013	China	219	21.0%	FT	Pyrosequencing
Liu et al. [23]	2017	China	210	22.9%	FFPE	Pyrosequencing
Baba et al. [18]	2015	Japan	440	23.0%	FFPE	Pyrosequencing
Yang et al. [31]	2017	China	24	4.2%	FFPE	NGS
Song et al. [28]	2014	China	158	4.5%	FT	NGS
Lin et al. [22]	2014	China	139	7.0%	FFPE	NGS
Gao et al. [19]	2014	Japan	133	9.0%	FT	NGS
Sawada et al. [26]	2016	Japan	144	10.4%	FT	NGS
TCGA et al. [9]	2017	Western and Eastern	90	13.0%	FT	NGS
Yokota et al. [37]	2018	Japan	126	13.5%	FFPE	NGS
Zhang et al. [32]	2015	China	90	17.0%	FT	NGS
Wang et al. [29]	2015	USA	71	24.0%	FFPE	NGS

NGS – next generation sequencing; FFPE – formalin-fixed paraffin-embedded tissue; FT – fresh frozen tissue

The *PIK3CA* gene is often mutated in several tumors types and most of its mutations occur in hotspot regions, such as E542K and E545A located in the helical domain (exon 9), and H1047R and H1047L in the kinase domain (exon 20) [11]. These mutations lead to the activation of the PIK3 pathway and have a great potential in oncogenic activities [11]. Interestingly, most of these mutations (E545A, H1047R and H1047L) occurred in patients in the CM/ESCC group and only one (E545A) in one patient in the ESCC group. We also identified other previously described important mutations (Table 3), the D549H mutation observed in the CM/ESCC group was reported in vulva and hepatocellular cancer [34]; R524K mutation found in the ESCC group was reported in colorectal cancer [35]; and the R555K mutation was reported in ovary cancer [36]. Interestingly, it is important to note that we identified three mutations in exon 20 that have not yet been reported (A1027D and K1030R in CM/ESCC group; T1053K in CM group). All these mutations occurred in patients with chagasic megaesophagus whose mutational profile of *PIK3CA* was never reported.

Importantly, we observed that CM/ESCC patients harboring *PIK3CA* mutations were associated with lower overall survival, suggesting its role as a prognostic biomarker in this group of patients. Interestingly, the results of our analyzes of the survival of the mutated patients differ from those reported by others studies, especially in regions of some risk such as Asia, in which patients with ESCC with mutations of the *PIK3CA* gene had a favorable overall survival compared to patients wild-type [37].

Notably, inhibitors of the PIK3-Akt-mTOR pathway have been developed as cancer target therapy alternatives, and patients harboring *PIK3CA* gene mutations could be potential candidates for such therapeutic approach [14]. Interestingly, phase I and II clinical trials using pan-*PIK3CA* agents (PIK3-class I), such as buparlisib (BKM120), an oral agent that affects α, β, γ and δ isoforms of PI3K [38], showed efficacy in several solid tumors, including head and neck cancer [39]. Copanlisib (BAY80–6946), an intravenous agent that affects α and δ isoforms of PI3K, also showed promising results in non-Hodgkin’s lymphomas [40]; as well as pictilisib (GDC-0941), an oral agent that affects γ and δ isoforms of PI3K, where a good response was reported in breast, colorectal, ovarian and non-small cell lung cancer [41]. Therefore, we can hypothesize that a subset of ESCC and CM/ESCC patients with *PIK3CA* mutations may benefit from these targeted-therapies and consequently improve their dismal survival.

In conclusion, this is the first study that analyzed and identified *PIK3CA* activating mutations in patients with esophageal squamous cell carcinomas associated with chagasic megaesophagus (CM/ESCC), which were associated with a worse outcome. Moreover, the identification of mutations in benign chagasic megaesophagus suggests their putative role in the etiology of esophageal squamous cell carcinoma and opens new opportunities for the treatment of these neglected patients with targeted-therapies.

## Abbreviations

CM: Chagasic megaesophagus
ESCC: Esophageal squamous cell carcinoma
FFPE: Formalin-fixed paraffin-embedded
MSI: Microsatellite instability
PCR: Polymerase chain reaction

## References

[1] Ferlay J, Soerjomataram I, Dikshit R, Eser S, Mathers C, Rebelo M, Parkin DM, Forman D, Bray F (2015). Cancer incidence and mortality worldwide: sources, methods and major patterns in GLOBOCAN 2012. Int J Cancer.

[2] Smyth EC, Lagergren J, Fitzgerald RC, Lordick F, Shah MA, Lagergren P, Cunningham D (2017). Oesophageal cancer. Nat Rev Dis Primers.

[3] Prabhu A, Obi KO, Rubenstein JH (2014). The synergistic effects of alcohol and tobacco consumption on the risk of esophageal squamous cell carcinoma: a meta-analysis. Am J Gastroenterol.

[4] Tustumi F, Bernardo WM, da Rocha JRM, Szachnowicz S, Seguro FC, Bianchi ET, Sallum RAA, Cecconello I (2017). Esophageal achalasia: a risk factor for carcinoma. A systematic review and meta-analysis. Dis Esophagus.

[5] Rassi A, Rassi A, Marin-Neto JA (2010). Chagas disease. Lancet.

[6] Pandolfino JE, Gawron AJ (2015). Achalasia: a systematic review. JAMA.

[7] Lacerda CF, Cruvinel-Carloni A, de Oliveira AT, Scapulatempo-Neto C, Lopez RV, Crema E, Adad SJ, Rodrigues MA, Henry MA, Guimaraes DP, Reis RM (2017). Mutational profile of TP53 in esophageal squamous cell carcinoma associated with chagasic megaesophagus. Dis Esophagus.

[8] Campanella NC, Lacerda CF, Berardinelli GN, Abrahao-Machado LF, Cruvinel-Carloni A, De Oliveira ATT, Scapulatempo-Neto C, Crema E, Adad SJ, Rodrigues MAM (2018). Presence of microsatellite instability in esophageal squamous cell carcinoma associated with chagasic megaesophagus. Biomark Med.

[9] Cancer Genome Atlas Research N, Analysis Working Group: Asan U, Agency BCC, Brigham, Women’s H, Broad I, Brown U, Case Western Reserve U, Dana-Farber Cancer I, Duke U (2017). Integrated genomic characterization of oesophageal carcinoma. Nature.

[10] Fruman DA, Chiu H, Hopkins BD, Bagrodia S, Cantley LC, Abraham RT (2017). The PI3K pathway in human disease. Cell.

[11] Karakas B, Bachman KE, Park BH (2006). Mutation of the PIK3CA oncogene in human cancers. Br J Cancer.

[12] Samuels Y, Wang Z, Bardelli A, Silliman N, Ptak J, Szabo S, Yan H, Gazdar A, Powell SM, Riggins GJ (2004). High frequency of mutations of the PIK3CA gene in human cancers. Science.

[13] Berenjeno IM, Pineiro R, Castillo SD, Pearce W, McGranahan N, Dewhurst SM, Meniel V, Birkbak NJ, Lau E, Sansregret L (2017). Oncogenic PIK3CA induces centrosome amplification and tolerance to genome doubling. Nat Commun.

[14] Luo J, Manning BD, Cantley LC (2003). Targeting the PI3K-Akt pathway in human cancer: rationale and promise. Cancer Cell.

[15] Velho S, Oliveira C, Ferreira A, Ferreira AC, Suriano G, Schwartz S, Duval A, Carneiro F, Machado JC, Hamelin R, Seruca R (2005). The prevalence of PIK3CA mutations in gastric and colon cancer. Eur J Cancer.

[16] World Health Organization. Neglected tropical diseases, hidden successes, emerging opportunities. Geneva: WHO Library Cataloguing-in-Publication; 2009.

[17] Akagi I, Miyashita M, Makino H, Nomura T, Hagiwara N, Takahashi K, Cho K, Mishima T, Ishibashi O, Ushijima T (2009). Overexpression of PIK3CA is associated with lymph node metastasis in esophageal squamous cell carcinoma. Int J Oncol.

[18] Baba Y, Ishimoto T, Harada K, Kosumi K, Murata A, Miyake K, Hiyoshi Y, Kurashige J, Iwatsuki M, Iwagami S (2015). Molecular characteristics of basaloid squamous cell carcinoma of the esophagus: analysis of KRAS, BRAF, and PIK3CA mutations and LINE-1 methylation. Ann Surg Oncol.

[19] Gao YB, Chen ZL, Li JG, Hu XD, Shi XJ, Sun ZM, Zhang F, Zhao ZR, Li ZT, Liu ZY (2014). Genetic landscape of esophageal squamous cell carcinoma. Nat Genet.

[20] Hou J, Jiang D, Zhang J, Gavine PR, Xu S, Liu Y, Xu C, Huang J, Tan Y, Wang H (2014). Frequency, characterization, and prognostic analysis of PIK3CA gene mutations in Chinese esophageal squamous cell carcinoma. Hum Pathol.

[21] Kim HS, Lee SE, Bae YS, Kim DJ, Lee CG, Hur J, Chung H, Park JC, Shin SK, Lee SK (2016). PIK3CA amplification is associated with poor prognosis among patients with curatively resected esophageal squamous cell carcinoma. Oncotarget.

[22] Lin DC, Hao JJ, Nagata Y, Xu L, Shang L, Meng X, Sato Y, Okuno Y, Varela AM, Ding LW (2014). Genomic and molecular characterization of esophageal squamous cell carcinoma. Nat Genet.

[23] Liu SY, Chen W, Chughtai EA, Qiao Z, Jiang JT, Li SM, Zhang W, Zhang J (2017). PIK3CA gene mutations in northwest Chinese esophageal squamous cell carcinoma. World J Gastroenterol.

[24] Mori R, Ishiguro H, Kimura M, Mitsui A, Sasaki H, Tomoda K, Mori Y, Ogawa R, Katada T, Kawano O (2008). PIK3CA mutation status in Japanese esophageal squamous cell carcinoma. J Surg Res.

[25] Phillips WA, Russell SE, Ciavarella ML, Choong DY, Montgomery KG, Smith K, Pearson RB, Thomas RJ, Campbell IG (2006). Mutation analysis of PIK3CA and PIK3CB in esophageal cancer and Barrett's esophagus. Int J Cancer.

[26] Sawada G, Niida A, Uchi R, Hirata H, Shimamura T, Suzuki Y, Shiraishi Y, Chiba K, Imoto S, Takahashi Y (2016). Genomic landscape of esophageal squamous cell carcinoma in a Japanese population. Gastroenterology.

[27] Shigaki H, Baba Y, Watanabe M, Murata A, Ishimoto T, Iwatsuki M, Iwagami S, Nosho K, Baba H (2013). PIK3CA mutation is associated with a favorable prognosis among patients with curatively resected esophageal squamous cell carcinoma. Clin Cancer Res.

[28] Song Y, Li L, Ou Y, Gao Z, Li E, Li X, Zhang W, Wang J, Xu L, Zhou Y (2014). Identification of genomic alterations in oesophageal squamous cell cancer. Nature.

[29] Wang K, Johnson A, Ali SM, Klempner SJ, Bekaii-Saab T, Vacirca JL, Khaira D, Yelensky R, Chmielecki J, Elvin JA (2015). Comprehensive genomic profiling of advanced esophageal squamous cell carcinomas and esophageal adenocarcinomas reveals similarities and differences. Oncologist.

[30] Wang WF, Xie Y, Zhou ZH, Qin ZH, Wu JC, He JK (2013). PIK3CA hypomethylation plays a key role in activation of the PI3K/AKT pathway in esophageal cancer in Chinese patients. Acta Pharmacol Sin.

[31] Yang JW, Choi YL (2017). Genomic profiling of esophageal squamous cell carcinoma (ESCC)-basis for precision medicine. Pathol Res Pract.

[32] Zhang L, Zhou Y, Cheng C, Cui H, Cheng L, Kong P, Wang J, Li Y, Chen W, Song B (2015). Genomic analyses reveal mutational signatures and frequently altered genes in esophageal squamous cell carcinoma. Am J Hum Genet.

[33] Zheng H, Wang Y, Tang C, Jones L, Ye H, Zhang G, Cao W, Li J, Liu L, Liu Z (2016). TP53, PIK3CA, FBXW7 and KRAS mutations in esophageal Cancer identified by targeted sequencing. Cancer Genomics Proteomics.

[34] Watkins JC, Howitt BE, Horowitz NS, Ritterhouse LL, Dong F, MacConaill LE, Garcia E, Lindeman NI, Lee LJ, Berkowitz RS (2017). Differentiated exophytic vulvar intraepithelial lesions are genetically distinct from keratinizing squamous cell carcinomas and contain mutations in PIK3CA. Mod Pathol.

[35] Berg M, Danielsen SA, Ahlquist T, Merok MA, Agesen TH, Vatn MH, Mala T, Sjo OH, Bakka A, Moberg I (2010). DNA sequence profiles of the colorectal cancer critical gene set KRAS-BRAF-PIK3CA-PTEN-TP53 related to age at disease onset. PLoS One.

[36] Niskakoski A, Kaur S, Renkonen-Sinisalo L, Lassus H, Jarvinen HJ, Mecklin JP, Butzow R, Peltomaki P (2013). Distinct molecular profiles in lynch syndrome-associated and sporadic ovarian carcinomas. Int J Cancer.

[37] Yokota T, Serizawa M, Hosokawa A, Kusafuka K, Mori K, Sugiyama T, Tsubosa Y, Koh Y (2018). PIK3CA mutation is a favorable prognostic factor in esophageal cancer: molecular profile by next-generation sequencing using surgically resected formalin-fixed, paraffin-embedded tissue. BMC Cancer.

[38] Maira SM, Pecchi S, Huang A, Burger M, Knapp M, Sterker D, Schnell C, Guthy D, Nagel T, Wiesmann M (2012). Identification and characterization of NVP-BKM120, an orally available pan-class I PI3-kinase inhibitor. Mol Cancer Ther.

[39] Soulieres D, Faivre S, Mesia R, Remenar E, Li SH, Karpenko A, Dechaphunkul A, Ochsenreither S, Kiss LA, Lin JC (2017). Buparlisib and paclitaxel in patients with platinum-pretreated recurrent or metastatic squamous cell carcinoma of the head and neck (BERIL-1): a randomised, double-blind, placebo-controlled phase 2 trial. Lancet Oncol.

[40] Patnaik A, Appleman LJ, Tolcher AW, Papadopoulos KP, Beeram M, Rasco DW, Weiss GJ, Sachdev JC, Chadha M, Fulk M (2016). First-in-human phase I study of copanlisib (BAY 80-6946), an intravenous pan-class I phosphatidylinositol 3-kinase inhibitor, in patients with advanced solid tumors and non-Hodgkin's lymphomas. Ann Oncol.

[41] Sarker D, Ang JE, Baird R, Kristeleit R, Shah K, Moreno V, Clarke PA, Raynaud FI, Levy G, Ware JA (2015). First-in-human phase I study of pictilisib (GDC-0941), a potent pan-class I phosphatidylinositol-3-kinase (PI3K) inhibitor, in patients with advanced solid tumors. Clin Cancer Res.

